# MicroRNA-based integrated diagnosis and therapy for GBM: current status and advances

**DOI:** 10.3389/fimmu.2026.1806864

**Published:** 2026-05-01

**Authors:** Ye Chen, Huiyi Liu, Peipei Yuan, Yongqi Li, Changqiong Xu, Ran Li, Jibing Chen, Fasheng Wu

**Affiliations:** 1 Guangxi University of Chinese Medicine, Nanning, China; 2 Ruikang Hospital Affiliated to Guangxi University of Chinese Medicine, Nanning, China; 3 Shaoguan University Medical College, Shaoguan, China; 4 Guangxi Academy of Sciences, Nanning, China; 5 Yichun University School of Medicine, Yichun, China

**Keywords:** biomarker, exosome, GBM, miRNA, therapeutic targets

## Abstract

Glioblastoma (GBM) is the most common and aggressive type of central nervous system cancer, characterized by high rates of recurrence and mortality. As the highest-grade glioma, patient prognosis remains poor despite multimodal interventions including surgery, chemotherapy, and postoperative radiotherapy. Therefore, developing novel therapeutic strategies and precise diagnostic tools has become an urgent need in oncology research. In recent years, exosomes have emerged as important candidates for targeted tumor therapy due to their natural, endogenous nanocarrier properties, such as low immunogenicity, good biocompatibility, and the ability to cross biological barriers. In particular, exosome-based delivery systems loading functional microRNAs (miRNAs) offer a promising new strategy for intervening in malignant tumor progression. Studies have shown that exosome-delivered tumor-suppressive miRNAs can effectively inhibit tumor cell proliferation, promote apoptosis, impede migration and invasion, and reverse chemoresistance. These functions have been validated through *in vitro* cellular models and *in vivo* animal experiments across various tumors, confirming the efficacy of engineered exosome-miRNA delivery systems in suppressing tumor growth, delaying metastasis, and sensitizing tumors to treatment. Furthermore, in the field of biomarkers, aberrant expression of various miRNAs is closely associated with GBM proliferation, invasion, metastasis, and therapy resistance. Specifically, downregulated tumor-suppressive miRNAs and upregulated oncogenic miRNAs may serve as potential biomarkers for monitoring disease progression, assessing prognosis, and predicting therapeutic response. In summary, the miRNA system offers dual potential as both a targeted therapeutic approach and a precise biomarker, providing new directions for the diagnosis and treatment of GBM. However, challenges such as optimizing delivery efficiency and enhancing targeting specificity remain. Moving forward, interdisciplinary efforts will be essential to overcome these technical barriers and advance its translation from basic research to clinical application.

## 1 Introduction

### 1.1 Clinical status and therapeutic challenges of GBM

Gliomas are the most common primary cancer of the central nervous system, originating from glial cells, and account for 40% to 60% of intracranial tumors. These tumors are characterized by rapid growth, high invasiveness, a tendency to recur, and resistance to treatment. Based on histological characteristics and clinical behavior, gliomas are classified into grades I to IV, where grade I and II are low-grade gliomas, mainly including diffuse astrocytoma band oligodendrogliomas. Grade III and IV are high-grade gliomas, with grade IV glioma, also known as GBM or GBM multiforme, being the most malignant type of brain glioma. In adult gliomas, GBM accounts for approximately 90%, making it the most common malignant glioma type (1).GBM is highly invasive and often rapidly infiltrates surrounding brain tissue and the spinal cord, making treatment extremely challenging (2). Epidemiological data indicate an annual incidence of 3.26 per 100,000 people, with a median overall survival of 15 months and a 5-year survival rate of only 6.9% (3). For GBM patients, overall survival is poor, and traditional therapies such as radiotherapy and chemotherapy offer limited benefits. Therefore, exploring new therapeutic strategies to overcome the current treatment bottleneck has become a critical issue in this field.

### 1.2The mechanisms underlying miRNA-based cancer therapy and detection techniques for miRNA biomarkers

MiRNAs are a class of small non-coding RNAs approximately 21–23 nucleotides in length. They primarily regulate gene expression at the post-transcriptional level by binding to the 3′-untranslated region (3′-UTR) of target miRNAs (4). MiRNAs are involved in a wide range of biological processes, including cell cycle regulation, proliferation, differentiation, and apoptosis, and play critical roles in tumor initiation and progression. In addition, miRNAs are stably present in body fluids, extracellular vesicles, and high-density lipoproteins, enabling their use as biomarkers for cancer detection, diagnosis, and prognostic evaluation (5).

At present, miRNA biomarker detection technologies have evolved into a multi-platform system. These primarily include nucleic acid amplification-based methods, such as stem-loop RT-PCR and rolling circle amplification, which enable highly sensitive detection of low-abundance miRNAs. Proprietary technologies, including ID3EAL™, can further achieve precise discrimination of homologous miRNAs differing by a single nucleotide. Nucleic acid hybridization-based microarray platforms are suitable for high-throughput screening, whereas biosensor technologies facilitate the development of rapid and point-of-care detection approaches (6).However, the high heterogeneity of the tumour microenvironment and the dynamic infiltration patterns of immune cells place greater demands on the precise classification of biomarkers. Recent studies have shown that integrating multi-omics technologies with frameworks for analysing the tumour microenvironment can reveal the associations between molecular biomarkers and immune status in a more systematic manner, thereby providing new strategies for the screening and validation of miRNA biomarkers (7).

### 1.3 Dual roles of miRNAs in GBM and the scope of the review

GBM, the most aggressive primary brain tumor, is characterized by profound dysregulation of miRNA networks during tumor progression. MiRNAs exert dual and context-dependent roles in GBM by functioning as either oncogenic or tumor-suppressive regulators, and disruption of this balance represents a central mechanism underlying GBM pathogenesis. This review summarises the current evidence regarding miRNAs as biomarkers for the growth, proliferation, metastasis and treatment resistance of glioblastoma, with a particular focus on miRNAs that have been validated through *in vitro* and *in vivo* experiments over the past five years and have been shown to have therapeutic effects on glioblastoma. Furthermore, we highlight the therapeutic potential of miRNAs as standalone or adjunctive antitumor agents for GBM, with broader implications for the development of novel treatments for neurological disorders. Notably, miRNA-based strategies also hold promise in the therapeutic landscape of other diseases.

### 1.4 Key advantages of exosomes as miRNA delivery vehicles

Exosomes are extracellular vesicles with diameters ranging from 30 to 150 nm and are characterized by a lipid bilayer membrane that closely resembles that of their parental cells. They are capable of crossing multiple physiological barriers and mediating intercellular communication through the transfer of bioactive molecules. Crucially, exosomes derived from GBM can cross the blood-brain barrier(BBB) and enter the peripheral blood, providing a unique molecular window for non-invasive liquid biopsy of central nervous system tumours.As delivery vehicles for miRNAs, exosomes effectively protect miRNAs from extracellular RNase-mediated degradation and facilitate their transport to adjacent or distant recipient cells (8), where they regulate target gene expression (9).Unlike circulating free miRNAs, which are readily degraded by nucleases, the lipid bilayer membrane structure of exosomes provides a physical barrier for the miRNAs contained within, ensuring their high stability in body fluids and thereby significantly enhancing the diagnostic sensitivity of liquid biopsy (10).

Among the various sources, exosomes derived from mesenchymal stem cells (MSCs) are the most extensively investigated in tumor therapy. These exosomes exhibit low immunogenicity and are well suited for local administration (11). In addition, they display strong tissue penetration capacity, enabling deep distribution across the epidermal, dermal, and subcutaneous layers (12). Importantly, MSC-derived exosomes retain functional characteristics of their parental cells, including innate tropism toward inflammatory sites. Through interactions with inflammatory cells, apoptotic cells, vascular endothelial cells, and tissue-resident stem cells, MSC-derived exosomes can modulate cellular behavior and reshape the tumor microenvironment, highlighting their translational potential in GBM therapy and immunomodulation (13).

## 2 MiRNA as biomarkers for GBM

### 2.1 MiRNAs downregulated in GBM

During the development of GBM, the miRNA expression profile undergoes significant remodeling. Notably, a class of tumour-suppressor miRNAs is consistently downregulated in GBM tissue; the loss of their function leads to a reduction in the suppression of oncogenic signaling pathways, thereby driving the malignant progression of the tumour. Studies have shown that the downregulation of various tumour-suppressive miRNAs in GBM is closely associated with poor patient prognosis. Taking miR-590-3p as an example, it is significantly underexpressed in GBM and exerts its tumour-suppressive effects by targeting multiple nodes in the SMAD2/3 pathway downstream of TGFBR2; restoring its expression can effectively inhibit tumour growth. Current research has identified a lot underexpressed miRNAs that have been validated through *in vitro* or *in vivo* experiments as having an impact on GBM (**Table 1**).

**Table 1:** List of downregulated miRNA in GBM.

Signaling pathway	MiRNA	Key target genes	Expression in GBM	Functional class	Biological functions	Evidence strength	Reference
PI3K/AKT/ mTOR	miR-4524b-5p	ALDH1A3	Down	Tumor suppressor	proliferation (-), Radiotherapy sensitivity (-)	High (*In vitro* and *in vivo*)	(14)
	miR-433	ERBB4	Down	Tumor suppressor	proliferation (-), Metastasis (-)	High (*In vitro* and *in vivo*)	(15)
	miR-219-5p	EGFR	Down	Tumor suppressor	proliferation (-)	Moderate (*In vitro*)	(16)
	miR-1	FN1	Down	Tumor suppressor	proliferation (-), Metastasis (-), TMZ sensitivity (+)	High (*In vitro* and *in vivo*)	(17)
	128	EGFR	Down	Tumor suppressor	proliferation (-)	Moderate (*In vitro*)	(16)
	miR-7	STX17, SNAP29	Down	Tumor suppressor	proliferation (-), Metastasis (-)	Moderate (*In vitro*)	(24)
	miR-1231	EGFR	Down	Tumor suppressor	proliferation (-)	High (*In vitro* and *in vivo*)	(19)
	miR-491	EGFR, CDK6, Bcl-xL	Down	Tumor suppressor	proliferation (-)	High (*In vitro* and *in vivo*)	(20)
	miR-200a-3p	GNAI1	Down	Tumor suppressor	proliferation (-)	High (*In vitro* and *in vivo*)	(21)
	miR-29	SCAP, SREBP-1	Down	Tumor suppressor	proliferation (-)	High (*In vitro* and *in vivo*)	(22)
	miR-374b	EGFR	Down	Tumor suppressor	proliferation (-), Metastasis (-)	Moderate (*In vitro*)	(23)
	miR-133	EGFR	Down	Tumor suppressor	Proliferation (-)	Moderate (*In vitro*)	(24)
	miR-340	ROCK1	Down	Tumor suppressor	proliferation (-), Metastasis (-)	Moderate (*In vitro*)	(25)
	miR-615	EGFR	Down	Tumor suppressor	proliferation (-), Metastasis (-)	High (*In vitro* and *in vivo*)	(26)
	miR-219-5p	EGFR	Down	Tumor suppressor	proliferation (-), Metastasis (-)	Moderate (*In vitro*)	(27)
	miR-146b-5p	EGFR	Down	Tumor suppressor	proliferation (-), Metastasis (-)	High (*In vitro* and *in vivo*)	(28)
	miR-373-3p	EGFR	Down	Tumor suppressor	proliferation (-), Metastasis (-)	Moderate (*In vitro*)	(29)
	miR-137	p-Akt, NF-κB, VEGF, b-FGF, EGFR, MMP-2/9, Bax/Bcl-2	Down	Tumor suppressor	proliferation (-), Metastasis (-)	Moderate (*In vitro*)	(30)
	miR-1298-5p	SETD7	Down	Tumor suppressor	proliferation (-)	High (*In vitro* and *in vivo*)	(31)
	miR-7	IRS-1, IRS-2	Down	Tumor suppressor	proliferation (-), TMZ sensitivity (+)	Moderate (*In vitro*)	(32)
	miR-181a	AKT1, Bcl-2, SIRT1, Caspase-9	Down	Tumor suppressor	proliferation (-), Metastasis (-), TMZ sensitivity (+)	Moderate (*In vitro*)	(33)
	miR-548x, miR-4698	PDK1, RHEB, AKT1, mTOR	Down	Tumor suppressor	proliferation (-)	Moderate (*In vitro*)	(34)
	miR-424	AKT1, RAF1	Down	Tumor suppressor	proliferation (-), Metastasis (-)	Moderate (*In vitro*)	(35)
	miR-489-3p	BDNF	Down	Tumor suppressor	proliferation (-), Metastasis (-)	Moderate (*In vitro*)	(36)
	miR-302a	PIK3CA, AKT, CXCR4, EGFR, LDHA, PFKP, ALDOB, ABCG2, EPHA2	Down	Tumor suppressor	proliferation (-), Metastasis (-), TMZ sensitivity (+)	Moderate (*In vitro*)	(37)
p53	miR-100	PLK1	Down	Tumor suppressor	proliferation (-), TMZ sensitivity (+)	High (*In vitro* and *in vivo*)	(38)
	miR-34a (1)	BCL2, SIRT1, SOX2, CASP7, CDK6	Down	Tumor suppressor	proliferation (-), TMZ sensitivity (+)	High (*In vitro* and *in vivo*)	(39)
	miR-490	TERF2, TNKS2, SMG1	Down	Tumor suppressor	proliferation (-)	Moderate (*In vitro*)	(40)
	miR-16-5p	p53	Down	Tumor suppressor	proliferation (-), Metastasis (-)	Moderate (*In vitro*)	(41)
	miR-29b, miR-125a	PDPN	Down	Tumor suppressor	proliferation (-), Metastasis (-)	Moderate (*In vitro*)	(42)
	miR-129	CDK4, CDK6, MDM2	Down	Tumor suppressor	proliferation (-), Metastasis (-)	Moderate (*In vitro*)	(43)
	miR-3928	MDM2, CD44, DDX3X, HMGA2, CCND1, BRAF, ATOH8, BMI1	Down	Tumor suppressor	proliferation (-), Metastasis (-)	High (*In vitro* and *in vivo*)	(44)
	miR-517c	KPNA2	Down	Tumor suppressor	proliferation (-), Metastasis (-)	High (*In vitro* and *in vivo*)	(45)
Wnt/β-catenin	miR-637	ZEB2, WNT7A, β-catenin, Cyclin D1	Down	Tumor suppressor	proliferation (-), Metastasis (-)	High (*In vitro* and *in vivo*)	(46)
	miR-566	VHL	Down	Tumor suppressor	proliferation (-), Metastasis (-), TMZ sensitivity (+)	High (*In vitro* and *in vivo*)	(47)
	miR-770	CDK8	Down	Tumor suppressor	proliferation (-)	Moderate (*In vitro*)	(48)
	miR-497-5p	RSPO2	Down	Tumor suppressor	proliferation (-), Metastasis (-)	High (*In vitro* and *in vivo*)	(49)
	miR-381	LEF1	Down	Tumor suppressor	Metastasis (-)	Moderate (*In vitro*)	(50)
	miR-101	SOX9	Down	Tumor suppressor	proliferation (-), Metastasis (-)	High (*In vitro* and *in vivo*)	(51)
	miR-137	LRP6	Down	Tumor suppressor	proliferation (-), Metastasis (-), TMZ sensitivity (+)	High (*In vitro* and *in vivo*)	(52)
	miR-126-3p	SOX2	Down	Tumor suppressor	proliferation (-), TMZ sensitivity (+)	Moderate (*In vitro*)	(53)
	miR-449b-5p	WNT2B	Down	Tumor suppressor	proliferation (-)	Moderate (*In vitro*)	(54)
	miR-206	FZD7	Down	Tumor suppressor	proliferation (-), Metastasis (-)	High (*In vitro* and *in vivo*)	(55)
	miR-23b	VHL	Down	Tumor suppressor	proliferation (-), Metastasis (-)	High (*In vitro* and *in vivo*)	(56)
	miR-935	FZD6	Down	Tumor suppressor	proliferation (-)	High (*In vitro* and *in vivo*)	(57)
Notch	miR-34c-3p, miR-34c-5p	Notch2	Down	Tumor suppressor	proliferation (-), Metastasis (-)	Moderate (*In vitro*)	(58)
	miR-326	Notch1, Notch2	Down	Tumor suppressor	proliferation (-), Metastasis (-)	Moderate (*In vitro*)	(59)
	miR-139-5p	Notch1	Down	Tumor suppressor	proliferation (-), Metastasis (-)	High (*In vitro* and *in vivo*)	(60)
MAPK/Erk	miR-134	KRAS, STAT5B	Down	Tumor suppressor	proliferation (-)	High (*In vitro* and *in vivo*)	(61)
	miR-145	ADAM17	Down	Tumor suppressor	proliferation (-), Metastasis (-)	Moderate (*In vitro*)	(62)
	miR-485-3p	RNF135	Down	Tumor suppressor	proliferation (-), Metastasis (-)	Moderate (*In vitro*)	(63)
NF-κB	miR-451	IKKβ	Down	Tumor suppressor	proliferation (-), Metastasis (-)	High (*In vitro* and *in vivo*)	(64)
	miR-155	AGTR1	Down	Tumor suppressor	proliferation (-), Metastasis (-), TMZ sensitivity (+)	High (*In vitro* and *in vivo*)	(65)
	miR-16	Bmi-1	Down	Tumor suppressor	proliferation (-), Metastasis (-)	High (*In vitro* and *in vivo*)	(66)
TGF-β	miR-590-3p	SMAD2/3	Down	Tumor suppressor	proliferation (-), TMZ sensitivity (+)	High (*In vitro* and *in vivo*)	(67)
	miR-564	TGF-β1	Down	Tumor suppressor	proliferation (-), Metastasis (-)	High (*In vitro* and *in vivo*)	(68)
	miR-524-3p, miR-524-5p	Smad2, Tead1, Hes1	Down	Tumor suppressor	proliferation (-), Metastasis (-)	High (*In vitro* and *in vivo*)	(69)
	miR-4286	TGFB1, TGFBR2	Down	Tumor suppressor	Metastasis (-)	Moderate (*In vitro*)	(70)
Regulation of EMT	miR-506	ETS1	Down	Tumor suppressor	proliferation (-), Metastasis (-), TMZ sensitivity (+)	High (*In vitro* and *in vivo*)	(71)
	miR-128-3p	IL-8	Down	Tumor suppressor	proliferation (-), Metastasis (-), TMZ sensitivity (+)	High (*In vitro* and *in vivo*)	(72)
	miR-365	PAX6	Down	Tumor suppressor	proliferation (-), Metastasis (-)	Moderate (*In vitro*)	(73)
	miR-378a-3p	IRG1	Down	Tumor suppressor	proliferation (-), Metastasis (-)	High (*In vitro* and *in vivo*)	(74)
	miR-200c	ZEB1	Down	Tumor suppressor	Metastasis (-)	Moderate (*In vitro*)	(75)
Regulation of the Cell Cycle and Apoptosis	miR-138	CD44, p27, AKT	Down	Tumor suppressor	proliferation (-), Metastasis (-)	High (*In vitro* and *in vivo*)	(76)
	miR-217-5p	EZH2, H3K27me3	Down	Tumor suppressor	proliferation (-), Metastasis (-), Radiotherapy sensitivity (-)	High (*In vitro* and *in vivo*)	(77)
	miR-646	p62/SQSTM1, Nrf2, HO-1	Down	Tumor suppressor	proliferation (-), Metastasis (-)	High (*In vitro* and *in vivo*)	(78)
	miR-1258	E2F1	Down	Tumor suppressor	proliferation (-), Metastasis (-), TMZ sensitivity (+)	High (*In vitro* and *in vivo*)	(79)
	miR-34a (2)	c-MET, CDK6, Notch1, Bcl-2	Down	Tumor suppressor	proliferation (-), Metastasis (-)	High (*In vitro* and *in vivo*)	(80)
	miR-181a	FBXO11, Rap1B, Bcl-2	Down	Tumor suppressor	proliferation (-)	High (*In vitro* and *in vivo*)	(81)
	miR-124 (3)	CDK4, CDK6, STAT3, NF-κB, PD-L1	Down	Tumor suppressor	proliferation (-), Metastasis (-)	High (*In vitro* and *in vivo*)	(82)
	miR-34a (3)	ATM, EGFR, BCL2, MET, UGCG	Down	Tumor suppressor	proliferation (-), TMZ sensitivity (+)	High (*In vitro* and *in vivo*)	(83)
	miR-758-5p	ZBTB20	Down	Tumor suppressor	proliferation (-), Metastasis (-)	Moderate (*In vitro*)	(84)
	miR-153	BCL2, Mcl-1, Nrf2	Down	Tumor suppressor	proliferation (-), Radiotherapy sensitivity (-)	High (*In vitro* and *in vivo*)	(85)
	miR-381	LRRC4	Down	Tumor suppressor	proliferation (-)	High (*In vitro* and *in vivo*)	(86)
	miR-4731	/	Down	Tumor suppressor	proliferation (-), Metastasis (-)	Moderate (*In vitro*)	(87)
	miR-520d-5p	PTTG1	Down	Tumor suppressor	proliferation (-)	High (*In vitro* and *in vivo*)	(88)
	miR-181a-5p	FBXO11	Down	Tumor suppressor	proliferation (-), Metastasis (-), TMZ sensitivity (+)	Moderate (*In vitro*)	(89)
	miR-211	MMP-9	Down	Tumor suppressor	proliferation (-), Metastasis (-), TMZ sensitivity (+), Radiotherapy sensitivity (-)	High (*In vitro* and *in vivo*)	(90)
	miR-342	BCL2L1, MCL1	Down	Tumor suppressor	proliferation (-)	Moderate (*In vitro*)	(91)
	miR-124-3p	RhoG	Down	Tumor suppressor	proliferation (-), Metastasis (-)	Moderate (*In vitro*)	(92)
	miR-7-1-3p	XIAP	Down	Tumor suppressor	proliferation (-), Metastasis (-)	High (*In vitro* and *in vivo*)	(93)
	miR-3174	CD44, MDM2, CDK6, RHOA, PLAU	Down	Tumor suppressor	proliferation (-), Metastasis (-), TMZ sensitivity (+)	High (*In vitro* and *in vivo*	(94)
Other pathways	miR-588	ROBO1, MMP2, MMP9	Down	Tumor suppressor	Metastasis (-)	High (*In vitro* and *in vivo*)	(95)
	miR-30c	SOX9	Down	Tumor suppressor	proliferation (-), Metastasis (-)	High (*In vitro* and *in vivo*)	(96)
	miR-124 (2)	Glt-1, Glast, xCT	Down	Tumor suppressor	proliferation (-)	High (*In vitro* and *in vivo*)	(97)
	miR-519a	STAT3	Down	Tumor suppressor	proliferation (-), TMZ sensitivity (+)	High (*In vitro* and *in vivo*)	(98)
	miR-125b	Bax/Bcl-2	Down	Tumor suppressor	proliferation (-), TMZ sensitivity (+)	Moderate (*In vitro*)	(99)
	miR-429	SOX2	Down	Tumor suppressor	proliferation (-), Metastasis (-)	Moderate (*In vitro*)	(100)
	miR-516a-5p	/	Down	Tumor suppressor	Metastasis (-)	Moderate (*In vitro*)	(101)
	miR-1268a	ABCC1, MRP1	Down	Tumor suppressor	proliferation (-), TMZ sensitivity (+)	High (*In vitro* and *in vivo*)	(102)
	miR-378a-3p	TSPAN17	Down	Tumor suppressor	proliferation (-), Metastasis (-)	Moderate (*In vitro*)	(74)
	miR-543	ADAM9	Down	Tumor suppressor	proliferation (-), Metastasis (-)	Moderate (*In vitro*)	(103)
	miR-376a	SIRT1	Down	Tumor suppressor	proliferation (-), Metastasis (-)	High (*In vitro* and *in vivo*)	(104)
	miR-576-3p	HIF-1α	Down	Tumor suppressor	proliferation (-), Metastasis (-)	Moderate (*In vitro*)	(105)
	miR-124a	IQGAP1, LAMC1, ITGB1	Down	Tumor suppressor	Metastasis (-)	Moderate (*In vitro*)	(106)
	miR-674-5p	Cul4b	Down	Tumor suppressor	proliferation (-), Metastasis (-)	High (*In vitro* and *in vivo*)	(107)
	miR-383	VEGF	Down	Tumor suppressor	proliferation (-), Metastasis (-)	Moderate (*In vitro*)	(108)
	miR-125b	MAZ	Down	Tumor suppressor	proliferation (-), Metastasis (-)	Moderate (*In vitro*)	(109)
	miR-9	ANXA2P2	Down	Tumor suppressor	proliferation (-)	Moderate (*In vitro*)	(110)
	miR-17	ATG7	Down	Tumor suppressor	TMZ sensitivity (+)	Moderate (*In vitro*)	(111)
	miR-30a	beclin 1	Down	Tumor suppressor	TMZ sensitivity (+)	Moderate (*In vitro*)	(112)
Regulation of DNA Damage and Repair	miR-124 (1)	RAD51	Down	Tumor suppressor	TMZ sensitivity (+)	High (*In vitro* and *in vivo*)	(113)
	miR-603, miR-221	MGMT	Down	Tumor suppressor	TMZ sensitivity (+)	High (*In vitro* and *in vivo*)	(114)
	miR-181	MGMT	Down	Tumor suppressor	TMZ sensitivity (+)	Moderate (*In vitro*)	(115)
	miR-198	MGMT	Down	Tumor suppressor	proliferation (-), TMZ sensitivity (+)	High (*In vitro* and *in vivo*)	(116)
	miR-203	ATM, RAD51	Down	Tumor suppressor	TMZ sensitivity (+)	Moderate (*In vitro*)	(117)
Glucose Metabolic	miR-3189	GLUT3	Down	Tumor suppressor	proliferation (-)	High (*In vitro* and *in vivo*)	(118)
	miR-106a	SLC2A3	Down	Tumor suppressor	proliferation (-)	Moderate (*In vitro*)	(119)
	miR-495	Glut1	Down	Tumor suppressor	proliferation (-)	Moderate (*In vitro*)	(120)
EGFR	miR-135a	NHE9	Down	Tumor suppressor	proliferation (-), Metastasis (-)	Moderate (*In vitro*)	(121)
	miR-22	SIRT1	Down	Tumor suppressor	proliferation (-), Metastasis (-)	Moderate (*In vitro*)	(122)
	miR-133b, miR-146a	PDGFRB, EGFR	Down	Tumor suppressor	proliferation (-)	Moderate (*In vitro*)	(62)

### 2.2 MiRNAs upregulated in GBM

During the development and progression of GBM, certain miRNAs exhibit significantly elevated expression. These upregulated miRNAs typically exert oncogenic effects and are extensively involved in several key processes, including tumour cell proliferation, invasion, treatment resistance and the remodeling of the immune microenvironment. Among these, miR-21 is currently one of the most extensively studied oncogenic miRNAs with the highest level of evidence. At the mechanistic level, miR-21 promotes tumour cell survival by inhibiting the PTEN gene and contributes to the development of resistance to radiotherapy and chemotherapy. Several studies have found that many miRNAs are highly expressed in GBM and play a promoting role in the disease (**Table 2**).

**Table 2:** List of upregulated miRNA in GBM.

Signaling pathway	MiRNA	Key target genes	Expression in GBM	Functional class	Biological functions	Evidence strength	Reference
PI3K/AKT /mTOR	miR-21	PTEN, PDCD4	Up	oncomiR	proliferation (+), Metastasis (+), TMZ sensitivity (-)	High (*In vitro* and *in vivo*)	(38, 123–125)
	miR-26a	PTEN	Up	oncomiR	proliferation (+)	Moderate (*In vitro*)	(126)
	miR-183	NEFL	Up	oncomiR	proliferation (+), Metastasis (+)	Moderate (*In vitro*)	(127)
	miR-1238	CAV1	Up	oncomiR	TMZ sensitivity (-)	High (*In vitro* and *in vivo*)	(128)
	miR-494	p190B, RhoGAP	Up	oncomiR	Metastasis (+)	Moderate (*In vitro*)	(129)
	miR-126	IRS1	Up	oncomiR	proliferation (+)	High (*In vitro* and *in vivo*)	(130)
	miR-15a-5p	CADM1	Up	oncomiR	proliferation (+), Metastasis (+)	Moderate (*In vitro*)	(131)
	miR-10b	AKT	Up	oncomiR	proliferation (+), Metastasis (+), TMZ sensitivity (-)	Moderate (*In vitro*)	(132)
p53	miR-3154	TP53INP1	Up	oncomiR	proliferation (+), Metastasis (+)	High (*In vitro* and *in vivo*)	(133)
	miR-141-3p	p53	Up	oncomiR	proliferation (+), TMZ sensitivity (-)	High (*In vitro* and *in vivo*)	(134)
Wnt/β-catenin	miR-296-3p	ICAT	Up	oncomiR	proliferation (+)	Moderate (*In vitro*)	(135)
	miR-133a	TGFBR1	Up	oncomiR	proliferation (+), Metastasis (+)	Moderate (*In vitro*)	(136)
	miR-301a	TCEAL7	Up	oncomiR	proliferation (+), TMZ sensitivity (-)	High (*In vitro* and *in vivo*)	(137)
MAPK/Erk	miR-21	PTEN, PDCD4, HIF-1α, VEGF	Up	oncomiR	proliferation (+)	High (*In vitro* and *in vivo*)	(138)
NF-κB	miR-17-92	TGFBR2, SMAD4	Up	oncomiR	proliferation (+)	High (*In vitro* and *in vivo*)	(139)
	miR-19a/b	SEPT7	Down	Tumor suppressor	proliferation (+), Metastasis (+)	High (*In vitro* and *in vivo*)	(140)
	miR-10a	RORA	Up	oncomiR	proliferation (+)	High (*In vitro* and *in vivo*)	(141)
	miR-221-3p, miR-22-3p	CHD7	Up	oncomiR	proliferation (+), TMZ sensitivity (-)	High (*In vitro* and *in vivo*)	(142)
TGF-β	miR-24	/	Up	oncomiR	proliferation (+)	Moderate (*In vitro*)	(143)
Regulation of EMT	miR-151a-3p	PDE4D	Down	Tumor suppressor	proliferation (+), Metastasis (+)	High (*In vitro* and *in vivo*)	(144)
Regulation of the Cell Cycle and Apoptosis	miR-21	TIMP3, RECK, PDCD4, PTEN, TAp63, SMAD7, BAX, BCL2, CDKN1A, CDC25A, BCL11B	Up	oncomiR	proliferation (+), Metastasis (+)	High (*In vitro* and *in vivo*)	(145)
	miR-21-5p	NEGR1, ANKS1B	Up	oncomiR	proliferation (+), Metastasis (+)	Moderate (*In vitro*)	(146)
	miR-339-5p	TUSC3	Up	oncomiR	proliferation (+), Metastasis (+)	Moderate (*In vitro*)	(146)
	miR-595	SOX7	Up	oncomiR	proliferation (+)	Moderate (*In vitro*)	(147)
	miR-296-5p	CASP8, NGFR	Up	oncomiR	proliferation (+), Metastasis (+)	Moderate (*In vitro*)	(148)
	miR-155-3p	Six1	Up	oncomiR	proliferation (+), TMZ sensitivity (-)	High (*In vitro* and *in vivo*)	(149)
	miR-93, miR-193	Cyclin D1	Up	oncomiR	proliferation (+), TMZ sensitivity (-)	High (*In vitro* and *in vivo*)	(150)
	miR-10b-5p	HOXB3	Up	oncomiR	proliferation (+), Metastasis (+)	Moderate (*In vitro*)	(151)
	miR-27a-3p	BTG2	Up	oncomiR	proliferation (+), TMZ sensitivity (-)	High (*In vitro* and *in vivo*)	(152)
Glucose Metabolic	miR-542-3p	HK2	Up	oncomiR	proliferation (+)	Moderate (*In vitro*)	(153)
Other pathways	miR-92b	FBXW7	Up	oncomiR	proliferation (+), Metastasis (+)	High (*In vitro* and *in vivo*)	(154)
	miR-23a	HOXD10	Up	oncomiR	proliferation (+), Metastasis (+)	Moderate (*In vitro*)	(155)
	miR-1246	FRK	Up	oncomiR	Metastasis (+)	Moderate (*In vitro*)	(156)
	miR-92	neogenin	Up	oncomiR	proliferation (+), Metastasis (+)	Moderate (*In vitro*)	(157)
	miR-92a	Prkar1a	Up	oncomiR	proliferation (+)	High (*In vitro* and *in vivo*)	(158)
	miR-191	NDST1	Up	oncomiR	proliferation (+)	High (*In vitro* and *in vivo*)	(159)
	miR-210-3p	Iscu	Up	oncomiR	proliferation (+), Metastasis (+)	High (*In vitro* and *in vivo*)	(160)
	miR-224-3p	ATG5	Up	oncomiR	proliferation (+), Metastasis (+), TMZ sensitivity (-)	High (*In vitro* and *in vivo*)	(161)

### 2.3 MiRNAs involved in multiple biological processes in GBM

**Figure 1:**
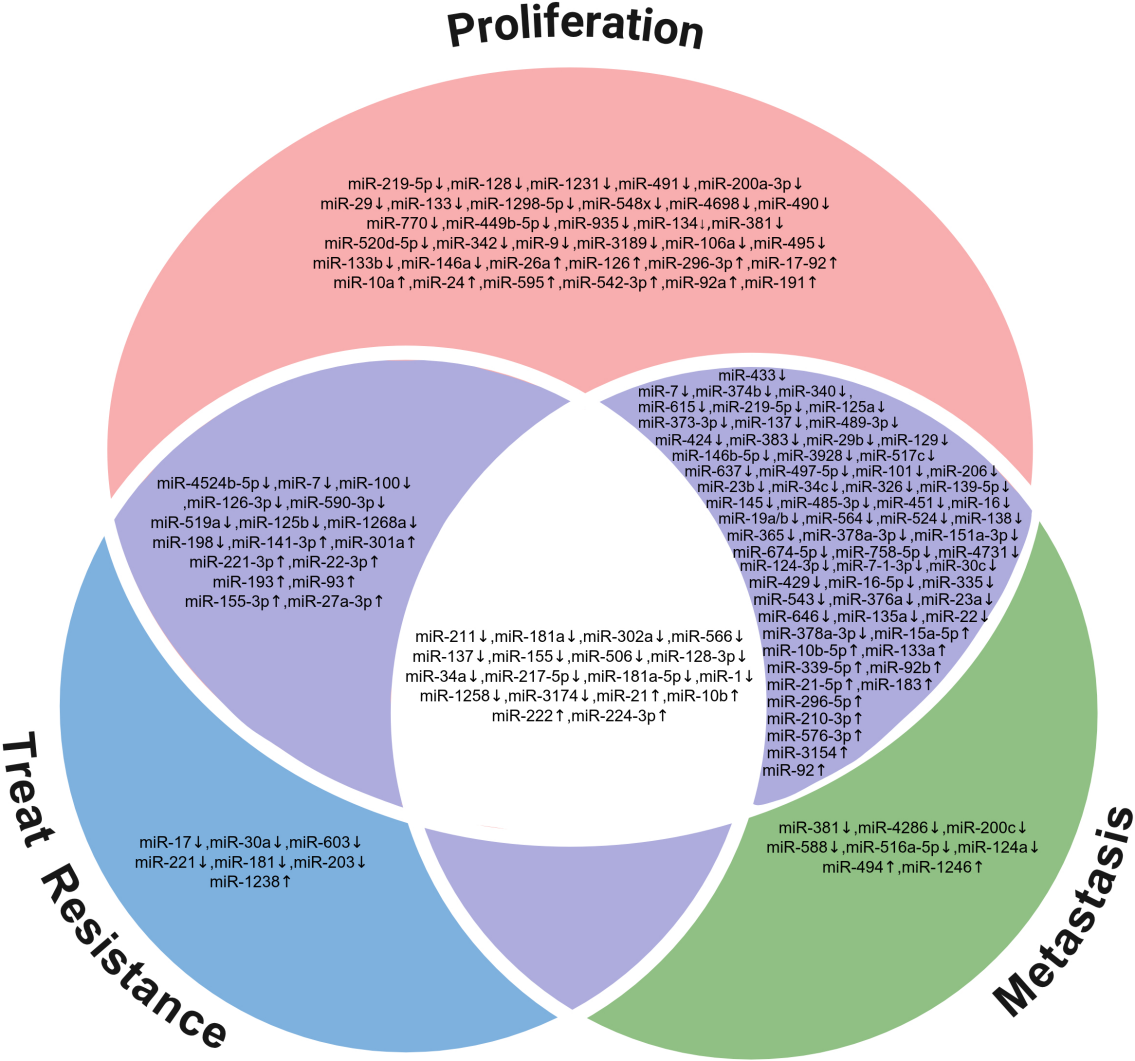
Role of miRNAs in GBM progression.

The malignant progression of glioblastoma is not driven by a single biological process, but rather results from the interplay and synergistic advancement of multiple factors, including proliferation, invasion and treatment resistance. In this process, certain miRNAs exhibit a ‘multi-target’ regulatory profile, acting simultaneously on several key signaling pathways to synergistically influence tumour cell growth, migration and drug resistance (**Figure 1**). For example, miR-34a, miR-21 and miR-124 have been shown to regulate the Notch, PI3K/Akt and Wnt/β-catenin pathways, respectively, thereby inhibiting proliferation whilst simultaneously reducing invasive potential and reversing treatment resistance. Such miRNAs with multi-regulatory functions not only provide a molecular hub for understanding the systemic regulatory network of GBM but also lay an important foundation for the development of multi-target combined intervention strategies.

## 3 Recent advances experimental research progress on miRNA-based therapy for GBM

**Figure 2:**
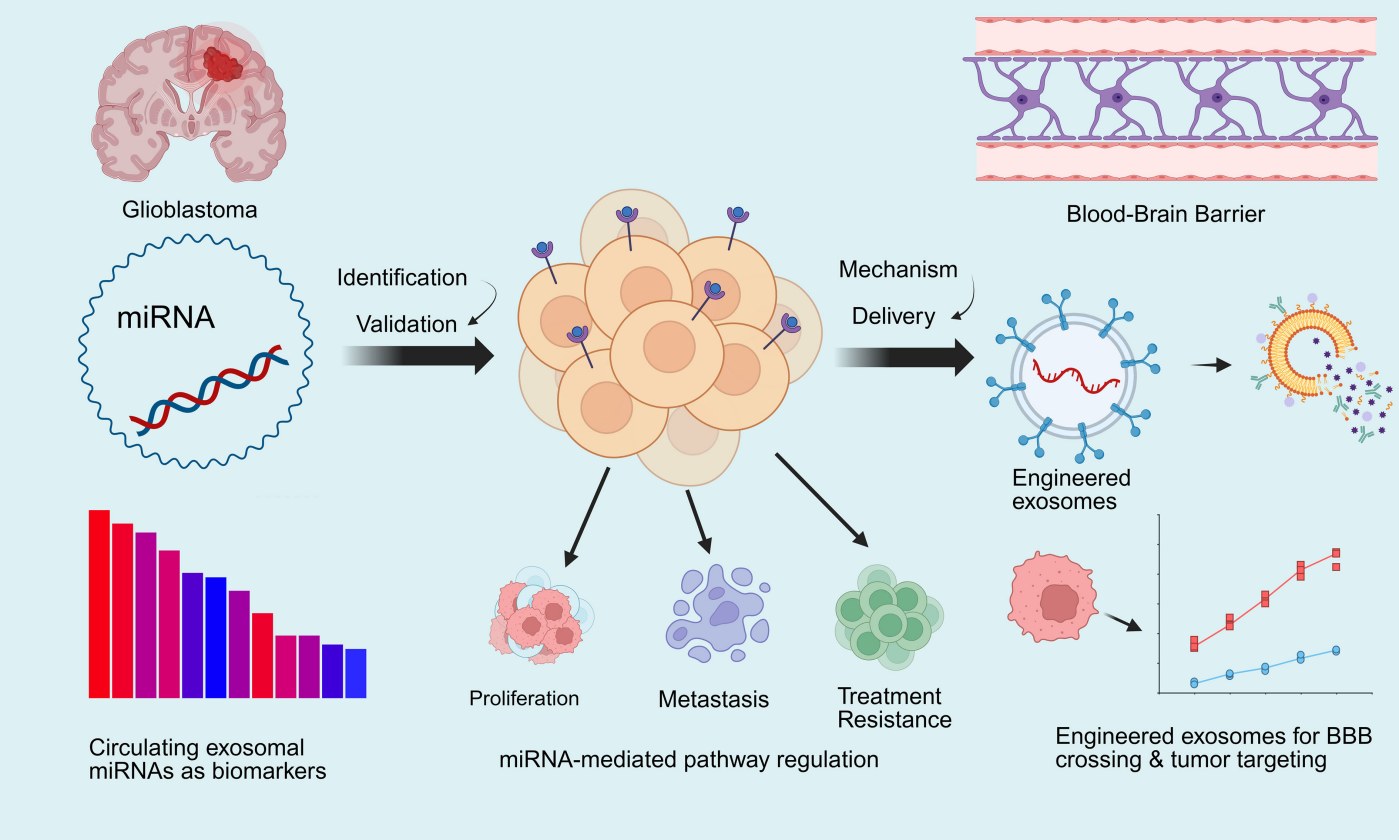
MicroRNAs involved in glioblastoma signaling pathways.

Based on the above data, and in order to systematically integrate and visualise the regulatory networks of these GBM-associated miRNAs validated by *in vitro* and *in vivo* experiments, we have further summarised the core mechanisms underlying exosome-mediated miRNA delivery for the treatment of GBM over the past five years (**Figure 2**). Among these, several tumour-suppressor miRNAs effectively inhibit GBM progression by targeting key oncogenes such as CDK6, EZH2, EGFR and IKKβ, thereby effectively arresting the cell cycle, inducing apoptosis and suppressing tumour stem cell properties; conversely, oncogenic miRNAs accelerate GBM progression and reduce sensitivity to chemotherapy and radiotherapy by inhibiting tumour-suppressor factors such as PTEN, PDCD4 and FBXW7.

### 3.1 Regulation of GBM proliferation

In terms of regulating tumour proliferation, several miRNAs inhibit GBM growth by targeting cell cycle-related proteins or key signaling pathways. For example, miR-138 (76)directly targets CD44, inducing nuclear translocation of p27, which leads to G0/G1 phase arrest; miR-637 (46) targets the WNT7A/β-catenin pathway, inhibiting cyclin D1 expression; miR-1258 (79) targets E2F1, thereby inhibiting PCNA transcription; miR-433 (15) targets ERBB4, inhibiting the PI3K/AKT pathway; miR-3174 (94) simultaneously targets CD44, MDM2 and RHOA, significantly inhibiting GBM cell proliferation and neurite formation. Furthermore, miR-7 (18) effectively inhibits tumour growth by blocking the late stages of autophagy by targeting STX17 and SNAP29 and disrupting mitochondrial function; miR-451 (64) targets the IKKβ/NF-κB pathway, inhibiting the expression of cyclin D1 and PCNA. However, there are also oncomiRs that drive GBM growth by targeting tumour suppressor genes or activating proliferation signaling pathways. miR-92b (154) is significantly upregulated in GBM and promotes cell proliferation and clonogenicity by directly targeting FBXW7; miR-19a/b (140) targets SEPT7 and activates the AKT/NF-κB pathway, upregulating cyclin D1 and PCNA to accelerate the G1/S phase transition; miR-151a-3p (144) is enriched in exosomes from hypoxic BTSCs; by activating the PDE4D enhancer in the nucleus, it upregulates the FAK/YAP signaling pathway, thereby driving GBM progression.

### 3.2 Regulation of GBM metastasis

In regulating tumour metastasis, miRNAs primarily exert their effects by targeting transcription factors and matrix metalloproteinases associated with the epithelial-mesenchymal transition (EMT). miR-588 (95) targets ROBO1, inhibiting hypoxia-induced MMP2/MMP9 expression and angiogenesis; miR-646 (78) targets the p62/Keap1/Nrf2 axis, upregulating E-cadherin and downregulating N-cadherin and Twist1; miR-506 (71) inhibits EMT via the FOXO1/miR-506/ETS1 feedback loop. Regarding the promotion of GBM metastasis, inhibition of miR-92b (154) reduces cell migration, whilst its overexpression enhances migratory capacity; miR-19a/b (140) promotes EMT by activating the Snail and Twist transcription factors, thereby downregulating E-cadherin and upregulating N-cadherin and vimentin; miR-151a-3p (144) induces EMT and cytoskeletal reorganisation via the PDE4D/FAK axis.

### 3.3 Reversal of targeted treatment resistance in GBM

In terms of overcoming treatment resistance, miRNAs sensitise temozolomide (TMZ) and radiotherapy by targeting mechanisms such as DNA damage repair, drug efflux and autophagy. Co-delivery of miR-603 and miR-221 targets MGMT, reversing TMZ resistance (114); miR-217-5p (77) targets EZH2, enhancing the efficacy of radiotherapy; miR-4524b-5p (14) targets ALDH1A3, overcoming radiotherapy resistance via the PI3K/AKT/mTOR pathway.miR-21 (38, 123–125), as a classic oncomiR, enhances TMZ resistance by targeting PTEN and PDCD4; its inhibition restores sensitivity to chemotherapy. These studies indicate that the combination of miRNAs with TMZ or radiotherapy exhibits synergistic antitumour effects, providing new strategies for the treatment of GBM.

### 3.4 miRNAs with multiple regulatory functions

Treatment resistance, abnormal proliferation, and invasion and metastasis in glioblastoma are often interrelated, and certain miRNAs can regulate these processes simultaneously.Such as, Studies have shown that miRNA-590-3p (67), miRNA-217-5p (77), miRNA-1258 (79) and miRNA-21 (38, 123–125, 138, 145) exhibit synergistic dysregulation in GBM and regulate mechanisms such as the cell cycle, apoptosis, epithelial-mesenchymal transition and DNA damage repair by acting on signaling pathways including Notch, PI3K/Akt and Wnt/β-catenin. *In vitro* and *in vivo* experiments have confirmed that modulating these miRNAs can simultaneously influence tumour growth, drug resistance and invasive capacity, suggesting their potential as multifunctional regulatory molecules and providing new targets and strategies for the combined treatment of GBM.

### 3.5 miRNA therapies entering the clinical trial phase

Although the preceding sections have systematically reviewed miRNAs that have demonstrated clear anti-GBM activity *in vitro* and *in vivo* experiments, it must be noted that the vast majority of studies remain at the preclinical stage, and only a very limited number of miRNA therapies have actually entered clinical trials (**Table 3**). This significant gap between basic research and clinical translation has become a key bottleneck constraining the development of miRNA therapeutics. Future research may focus on the diagnostic or therapeutic applications of microRNAs in clinical trials.

**Table 3:** Clinical trials of microRNAs for GBM.

Project	Title	Target	Diagnosis or Treatment	Status
NCT01829971	A multicenter phase I study of MRX34, microRNA miR-RX34 liposomal injection	miR-34a	Treatment	Terminated, for 5 immune-related serious adverse events
NCT05908773	A Microdose Study of TTX-MC138-NODAGA-Cu64 in Subjects With Advanced Solid Tumors	miR-10b	Diagnosis	Completed
NCT06260774	Study of TTX-MC138 in Subjects With Advanced Solid Tumors	miR-10b	Treatment	recruiting
NCT01849952	Evaluating the Expression Levels of MicroRNA-10b in Patients With Gliomas	miR-10b	Diagnosis	recruiting
NCT06203496	Monitoring of Patients With Diffuse Gliomas Using Circulating miRNAs (GliomiR)	Oncogenic miRNA clusters	Diagnosis	recruiting
NCT06883214	Three miRNA Signatures in Glioma: From Molecular Mechanisms to Potential Clinical Application	miR-1-3p, miR-26a-1-3p, miR-487b-3p	Diagnosis	recruiting

## 4 Discussion I: current challenges in the use of exosome-miRNA delivery systems for the diagnosis and treatment of GBM

### 4.1 Clinical translation of exosomes as delivery vehicles: challenges from preparation to delivery efficiency

Exosomes, as natural nanocarriers, show tremendous potential in the targeted delivery of miRNAs for GBM therapy and in liquid biopsy applications. However, their clinical translation faces three core bottlenecks. First, the standardization and scalability of exosome preparation methods remain key challenges. Current techniques such as ultracentrifugation and polymer precipitation suffer from issues like low yield, poor purity, and high heterogeneity (162). The functional differences among the obtained exosome subpopulations directly impact the reproducibility of subsequent research and therapeutic efficacy. Second, the active loading efficiency and stability of exogenous miRNAs urgently need improvement. Loading exogenous miRNAs via methods like electroporation (163)or chemical transfection can easily lead to aggregation or degradation. Furthermore, the natural targeting properties of exosomes may be altered after drug loading. Therefore, how to efficiently load miRNAs while maintaining their biological activity is a critical pain point in drug development. Finally, the precision and penetrability of the delivery system require breakthroughs. Even after successful loading, exosomes still need to cross the BBB and specifically target GBM cells while avoiding clearance by normal tissues (164). The current insufficient understanding of their homing mechanism limits the design of active targeting strategies. In summary, resolving the technical contradictions in the three key stages—preparation, loading, and delivery—is the core prerequisite for advancing exosome-miRNA therapy and diagnostic platforms towards clinical practice in GBM.

Furthermore, the delivery efficiency of exosomes in clinical treatment faces significant challenges, which constitutes a key bottleneck hindering their translation into clinical practice. Firstly, quantitative evidence regarding *in vivo* delivery efficiency remains extremely limited. Most current studies are confined to mouse xenograft models, with a lack of pharmacokinetic data from large animal models such as non-human primates. Existing research indicates that systemically administered unmodified exosomes are rapidly cleared by the mononuclear phagocyte system in the liver and spleen, with the proportion distributed within the brain typically accounting for less than 1% of the total administered dose. For intracranial tumours such as GBM, the actual efficiency of crossing the BBB and accumulating at the tumour site falls far short of the therapeutic threshold (165). Secondly, compared with mature delivery systems such as lipid nanoparticles (LNPs), the biodistribution of exosomes is less controllable. LNPs can precisely regulate their circulation half-life (ranging from several hours to tens of hours) through the proportion of ionisable lipids and surface PEGylation, whereas the half-life of native exosomes is typically only a few minutes to tens of minutes, making it difficult to support stable systemic dosing regimens (166). Thirdly, batch-to-batch heterogeneity represents a fundamental obstacle to the industrialisation of exosome-based therapeutics (167). Traditional separation methods, such as ultracentrifugation, yield low volumes and produce inconsistent purity; even when using tangential flow filtration or microfluidic technologies, significant variations persist across batches in terms of particle size distribution, membrane protein composition and miRNA loading efficiency (168), which falls far short of regulatory requirements for drug consistency.In addition to the aforementioned issues, the physical and biochemical barrier properties of the blood-brain barrier (BBB) itself constitute another key limitation. Even though exosomes possess an innate ability to cross the BBB, the loading of exogenous miRNAs may alter their surface protein composition and targeting properties, leading to a significant reduction in their efficiency of transendothelial transport (169). Furthermore, the efficiency of exosome penetration into the brain parenchyma and uptake by target cells following BBB crossing is similarly low, ultimately resulting in drug accumulation at tumour sites that falls far below the therapeutic threshold (170). Studies have shown that unmodified exosomes crossing the BBB rely primarily on passive transport or non-specific adsorption, making it difficult to achieve effective accumulation within GBM lesions (171).

In response to the aforementioned bottlenecks, engineered exosome strategies have gradually emerged in recent years as a key approach to enhancing delivery efficiency. By conjugating targeting ligands (such as transferrin, RGD peptides, or anti-EGFR nanobodies) to the surface of exosomes, active targeted accumulation can be achieved following crossing of the BBB; polymer coating or lipid hybridisation strategies can extend their circulation half-life, thereby avoiding rapid clearance by the mononuclear phagocyte system (172). It is worth noting that research progress in biomimetic and personalised delivery platforms has provided new insights for the design of exosome carriers. Strategies exemplified by biomimetic nanovaccines demonstrate that by mimicking the interfacial properties of natural biological particles, the *in vivo* stability and targeting specificity of delivery systems can be significantly enhanced; these design concepts hold significant implications for the optimisation of engineered exosomes (173). Furthermore, the integrated application of microfluidic technology not only holds promise for achieving high-purity, high-yield exosome separation but also enables real-time quality control of miRNA loading efficiency, providing a standardised pathway for the preparation of clinical-grade exosomes (174). In the future, the clinical translation of exosomes as miRNA delivery vehicles will depend not only on improvements in delivery efficiency itself, but also on simultaneous breakthroughs across three dimensions: pharmacokinetic characterisation, large-scale preparation processes, and quality control systems. Only then can the field progress from proof of concept to clinical validation.

### 4.2 Barriers to clinical translation of miRNAs

miRNAs demonstrate significant potential in therapeutic intervention and diagnostic biomarker development for GBM. However, their translation from basic research to clinical application still faces multiple intertwined scientific and technical obstacles, which together constitute the main bottlenecks along the translational pathway.

In terms of therapeutic mechanisms and clinical translation, the primary challenge lies in the off-target risks inherent to the multi-target effect of miRNAs (175). A single miRNA, through imperfect base pairing, can regulate dozens or even hundreds of target genes (176). While this network-level regulation disrupts key tumor pathways, it inevitably interferes with the physiological functions of normal cells, potentially leading to unpredictable toxic side effects. Second, the animal models relied upon in preclinical studies differ fundamentally from the complex human GBM context, which includes a distinctive immune microenvironment, cellular heterogeneity, and the specific structure of the BBB (177). This discrepancy means that miRNA therapies showing excellent efficacy in animal models may fail in humans due to insufficient delivery efficiency, immune clearance, or microenvironmental suppression, limiting the predictive value of preclinical data. Third, a standardized safety evaluation system specifically for nucleic acid drugs has not yet been established (178). The toxicological evaluation criteria for traditional small-molecule drugs are not directly applicable to miRNAs. The long-term immunogenicity, organ accumulation potential, and potential epigenetic impacts of miRNAs lack systematic and widely accepted assessment standards, introducing uncertainty into the design and regulatory review of clinical trials.

In the realm of diagnosis and biomarker application, the core obstacle is the insufficient specificity of a single miRNA as a biomarker (179). GBM exhibits diverse molecular subtypes, and miRNA expression can be influenced by systemic factors. Fluctuations in a single miRNA may originate from various pathological or physiological states, making it difficult to accurately distinguish tumor progression, treatment response, or inflammatory changes (180). Furthermore, the lack of standardised testing criteria, coupled with variations in sample processing and extraction methods, further compromises the comparability of results. Whilst multi-marker panel testing can improve diagnostic accuracy, it is relatively costly; moreover, most candidate markers lack large-scale, multicentre prospective clinical validation. Additionally, the limited availability of high-end testing equipment in primary care settings restricts the widespread adoption of these technologies.

In light of the aforementioned bottlenecks, future translational pathways must be advanced through a coordinated approach encompassing both mechanistic breakthroughs and systematic integration. At the therapeutic level, bioinformatics tools must be utilised to accurately predict and validate core target clusters, whilst developing smart responsive delivery systems (such as lipid nanoparticles and engineered exosomes) to enhance *in vivo* stability, BBB penetration and targeting specificity (166), and to establish a novel safety assessment paradigm tailored to nucleic acid therapeutics; At the diagnostic level, it is essential to advance research into biomarker panels based on multidimensional omics data. The use of miRNA panels comprising multiple functionally synergistic or signaling pathway-complementary miRNAs can more comprehensively reflect the characteristics of the tumour microenvironment and molecular subtypes, significantly improving diagnostic specificity and the accuracy of treatment response prediction (181), whilst strictly standardising the clinical validation process.

In summary, to advance the application of miRNAs in the field of GBM, a systematic translational strategy is required: this involves the seamless integration of highly sensitive detection technologies, multi-target miRNA panels and advanced delivery systems to form a closed-loop detectio-typing-intervention process (182). Only through multidisciplinary collaboration, by overcoming these interrelated obstacles one by one, can the theoretical potential of miRNAs ultimately be transformed into clinical tools capable of improving the prognosis of GBM patients.

### 4.3 Comparative analysis of miRNAs and other RNA therapeutics

In addition to miRNAs, therapeutic strategies targeting RNA or utilising it as an effector molecule also include small interfering RNA (siRNA), messenger RNA (mRNA) and CRISPR-based gene editing technologies. These RNA therapeutics each have distinct characteristics in terms of their mechanisms of action, delivery requirements, safety profiles and stage of clinical translation, creating a landscape in which they both complement and compete with miRNAs.

#### 4.3.1 Differences in mechanism of action

siRNA induces the degradation of target mRNA through perfect base pairing, thereby exerting a sequence-specific gene silencing effect. Although its targets are singular and highly predictable (183), in the case of tumours—which are multi-gene-driven diseases—inhibition of a single target often fails to achieve lasting therapeutic efficacy. In contrast, miRNAs regulate dozens to hundreds of target genes simultaneously through non-complementary base pairing, creating a networked regulatory effect. This multi-target nature offers unique advantages when intervening in the complex signaling pathway networks of tumours, but it also increases the risk of off-target effects and complicates the elucidation of mechanisms (184). mRNA therapies deliver mRNA encoding therapeutic proteins, which are translated within cells to produce functional proteins. They are suitable for protein replacement therapy or the development of tumour vaccines; however, they suffer from poor *in vivo* stability, high immunogenicity, and difficulty in achieving fine-tuned regulation of endogenous pathways within tumour cells (185). CRISPR technology enables permanent gene editing at the DNA level via Cas nucleases and guide RNAs (gRNAs), offering the potential for one-time therapy; however, safety risks associated with genomic integration, off-target editing and delivery efficiency remain major obstacles to clinical translation (186).

#### 4.3.2 Common characteristics of delivery vehicles

The aforementioned RNA therapeutics all face similar delivery bottlenecks, including poor *in vivo* stability, susceptibility to degradation by nucleases, immunogenicity, and challenges in targeted delivery to specific tissues (particularly across the BBB). Lipid nanoparticles (LNPs) are currently the most mature RNA delivery platform and have been successfully employed in clinical applications for COVID-19 mRNA vaccines and siRNA therapeutics (such as Onpattro^®^) (187). However, the efficiency of LNPs in targeting intracranial tumours remains suboptimal, and certain components pose risks of hepatotoxicity and immune activation (188). Exosomes, as endogenous nanocarriers, demonstrate unique potential for RNA delivery in GBM due to their low immunogenicity, good biocompatibility and natural ability to cross the BBB (189);however, their large-scale production and batch consistency remain key bottlenecks constraining clinical translation (190).

#### 4.3.3 Future trends in collaboration

It is worth noting that different RNA technologies are not mutually exclusive, but rather show a trend towards convergence and integration. For example, CRISPR technology can be used to achieve precise editing of endogenous miRNAs, systematically silencing oncogenic miRNAs or activating tumour-suppressing miRNAs through the delivery of gRNA and Cas proteins (191); mRNA therapy can encode tumour-suppressing factors that act in synergy with miRNAs; whilst engineered exosomes, as a universal delivery platform, hold promise for simultaneously carrying miRNAs, siRNAs or mRNAs, thereby enabling multimodal combination therapy (192). Consequently, in the treatment of GBM, the relationship between miRNA therapy and other RNA therapeutics is characterised more by complementary synergy than by substitution and competition. Future development should focus on selecting the most appropriate RNA intervention strategy based on molecular subtypes of the disease, whilst exploring the combined application of multiple RNA technologies.

### 4.4 Innovations in miRNA research driven by cutting-edge technologies: single-cell, spatial transcriptomics and artificial intelligence

The bottlenecks in the diagnosis and treatment of GBM described above are now being addressed through the integration of single-cell resolution and artificial intelligence technologies, which is paving the way for breakthrough solutions.

#### 4.4.1 miRNA regulatory heterogeneity at single-cell resolution

Traditional bulk RNA-seq masks the immense cellular heterogeneity within GBM. In recent years, miRNA research methods based on single-cell RNA sequencing (scRNA-seq) have made significant progress. For example, the PSCSR-seq V2 method enables the simultaneous sequencing of miRNAs and mRNAs within individual cells, allowing for the direct construction of cell-type-specific regulatory networks (193). Through scRNA-seq, researchers have discovered that the same miRNA may play entirely different roles in tumour cells, macrophages and T cells within GBM, promoting proliferation in tumour cells whilst potentially mediating immune suppression in macrophages (194). This cell-type-specific understanding is a prerequisite for screening highly specific biomarkers and developing precision-targeted therapies. Furthermore, computational methods such as micro-imp and miTEA-HiRes can directly infer single-cell miRNA activity from scRNA-seq data without the need for separate small RNA sequencing, greatly expanding the value of public data mining (195).

#### 4.4.2 Spatial transcriptomics reveals the spatial distribution of miRNAs

The malignant progression of GBM is highly dependent on the complex spatial architecture of its microenvironment. Spatial transcriptomics technologies (such as 10x Visium and STmiR) enable the mapping of miRNA and their target gene expression profiles *in situ* within tissue (196). The value of this technology lies in the following: firstly, it can identify miRNAs enriched in specific spatial niches, which are often closely associated with treatment resistance and invasiveness; secondly, it can visualise the spatial co-localisation of miRNAs with immune cells (such as tumour-associated macrophages), thereby identifying hotspots of intercellular communication. For exosomal miRNAs, spatial transcriptomics can even track the diffusion gradient of exosomes from the tumour core to the invasive front, providing direct evidence of spatial heterogeneity in delivery efficiency.

#### 4.4.3 AI-driven multimodal biomarker discovery and model development

Diagnostic and therapeutic decision-making for GBM is shifting from a single-marker approach to a multi-modal integrated model. AI, particularly deep learning, has a natural advantage in handling the high-dimensional, sparse and non-linear characteristics of scRNA-seq, spatial transcriptomics, imaging and clinical data. Specifically in the field of miRNAs, the application of AI manifests at three levels: Firstly, biomarker discovery, utilising graph neural networks to integrate miRNA-mRNA regulatory networks and automatically identify miRNA combinations with the highest diagnostic or prognostic value from single-cell data (197); Secondly, treatment response prediction, training models based on patients tumour tissue miRNA expression profiles and clinical characteristics to predict their sensitivity to temozolomide or specific miRNA therapies (198); Third, delivery system design: utilising machine learning to optimise the surface modification of exosomes and the composition of LNPs, thereby predicting their *in vivo* biodistribution and efficiency in crossing the BBB (199). Existing research has employed deep learning to predict miRNA targets from sequence information, and has successfully applied this to the screening of synthetic lethal targets in GBM.

### 4.5 Overview of integrated diagnostic and therapeutic approaches: from mechanisms to delivery to biomarkers

**Figure 3:**
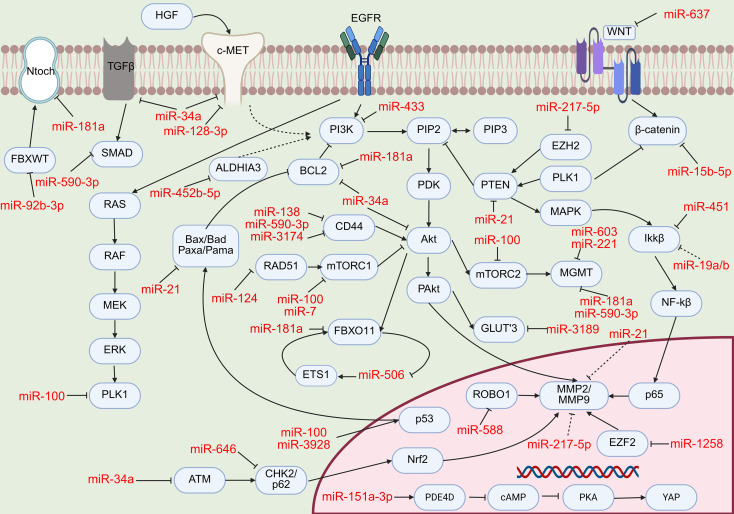
Translational pipeline for miRNA-based theranostics in GBM.

In summary, the application of miRNAs in the diagnosis and treatment of GBM encompasses two intertwined dimensions: firstly, as therapeutic targets, where engineered exosomes are used to deliver miRNAs across the blood-brain barrier to regulate tumour proliferation, metastasis and treatment resistance; and secondly, as liquid biopsy biomarkers, utilising circulating exosomal miRNAs to enable non-invasive diagnosis and dynamic monitoring. This provides a visual representation of the integrated diagnostic and therapeutic pathway encompassing mechanism elucidation, delivery intervention and biomarker application(**Figure 3**).

## 5 Discussion II: conclusion and future perspectives

### 5.1 Conclusions

GBM, as the most aggressive primary brain tumor, has long been limited in its treatment by high recurrence rates and therapeutic resistance. The efficacy of conventional therapies has reached a plateau, prompting research to seek new breakthroughs at the level of epigenetic regulation. MicroRNAs, a class of small non-coding RNAs, play a dual regulatory role in this context. This expression imbalance constitutes a sophisticated regulatory network, positioning miRNAs as highly promising biomarkers and therapeutic targets. From a diagnostic perspective, the stable miRNA expression profiles present in tissues or bodily fluids provide new tools for the non-invasive early diagnosis, molecular typing, and prognostic evaluation of GBM. From a therapeutic standpoint, adjusting their expression by delivering tumor-suppressive miRNA mimics or oncogenic miRNA inhibitors theoretically enables the multi-pathway synergistic inhibition of tumor progression.

### 5.2 Future outlook: research directions for clinical translation

To overcome current obstacles and propel miRNA research from the laboratory to clinical application, future work needs to focus on three synergistic and co-advancing pathways: technological innovation, in-depth mechanistic understanding, and clinical translation.

In terms of technological innovation, the development of next-generation smart delivery systems is required. This includes optimizing the standardized preparation (200), and engineered targeted modification of exosomes. Crucially, single-cell sequencing, spatial transcriptomics and artificial intelligence should be actively incorporated: utilising single-cell resolution to map cell-type-specific miRNA profiles in the GBM micro-environment; and employing spatial transcriptomics to reveal the spatial regulatory networks of this micro-environment; and utilising AI to construct multimodal data integration and predictive models to accelerate the screening of effective miRNA combinations from vast amounts of data and optimizing delivery strategies.

For mechanistic understanding, research must move beyond single-point knowledge. A systematic analysis of the global regulatory network of miRNAs should be conducted through multi-omics integration(particularly when combined with single-cell and spatial data). Focus should be placed on their role in maintaining tumor stem cell stemness and mediating therapeutic resistance, and elucidating their contribution to intercellular communication within the tumor microenvironment via extracellular vesicles.

In terms of clinical translation, there is an urgent need to utilise AI algorithms to construct multi-miRNA biomarker models based on large-scale, multi-centre samples analysed at single-cell resolution, and to establish a comprehensive standardised system covering everything from sample processing to data analysis. It is essential to advance early-stage clinical trials of miRNA-targeted therapies and to use AI-assisted design to optimizing trial protocols.
