# Correction: MicroRNA-based integrated diagnosis and therapy for GBM: current status and advances

**DOI:** 10.3389/fimmu.2026.1897131

**Published:** 2026-06-24

**Authors:** Ye Chen, Huiyi Liu, Peipei Yuan, Yongqi Li, Changqiong Xu, Ran Li, Jibing Chen, Fasheng Wu

**Affiliations:** 1 Guangxi University of Chinese Medicine, Nanning, China; 2 Ruikang Hospital Affiliated to Guangxi University of Chinese Medicine, Nanning, China; 3 Shaoguan University Medical College, Shaoguan, China; 4 Guangxi Academy of Sciences, Nanning, China; 5 Yichun University School of Medicine, Yichun, China

**Keywords:** biomarker, exosome, GBM, miRNA, therapeutic targets

In the published article, One funded project was omitted. The funder “Joint Project of Science and Technology Innovation of Jiangxi Provincial Health Commission, 2025L3081” to “Ran Li” was erroneously omitted.

The original version of this article has been updated.

An incorrect **Funding** statement was provided. The correct **Funding** statement reads: “The author(s) declared that financial support was received for this work and/or its publication. This work was supported by grants from the National Natural Science Foundation of China (No.82460763),Guangxi Youth Qihuang Scholars Training Program (GXQH202422), Natural Science Foundation of Guangdong province (No.2025A1515012507), Precision dosing strategies for CyberKnife treatment of intracranial lesions: a prospective cohort study based on anatomical-functional segmentation (Grant No. 2025YJ-34), Joint Project of Science and Technology Innovation of Jiangxi Provincial Health Commission(No.2025L3081), Shaoguan University doctoral research fund (No.9900064704).”

In the published article, there is a typographical error in the title of Section 2.2. The original title was 2.2 *MiRNAs downregulated in GBM*, whereas the section actually discusses miRNAs that are upregulated in GBM. The correct subsection title is 2.2 *MiRNAs upregulated in GBM*.

The original version of this article has been updated.

